# Span Value as a Critical Quality Attribute for PLGA Microspheres: Controlling Burst Release and Enhancing Therapeutic Efficacy via Wet Sieving

**DOI:** 10.3390/pharmaceutics18020180

**Published:** 2026-01-30

**Authors:** Lele Wang, Wenqiang Liu, Qiqi Jiang, Xin Wang, Dongdong Xu, Ying Fang, Simeng Wang, Jihui Tang

**Affiliations:** Research and Industrialization of New Drug Release Technology Joint Laboratory of Anhui Province, School of Pharmacy, Anhui Medical University, Hefei 230032, China; wanglele202202@163.com (L.W.); 2345010826@stu.ahmu.edu.cn (W.L.); 19855376356@163.com (Q.J.); wxin10262024@163.com (X.W.); 17719351016@163.com (D.X.); yingfang163163@163.com (Y.F.); 15855633143@163.com (S.W.)

**Keywords:** triamcinolone acetonide, particle size, polydispersity, knee osteoarthritis, pharmacokinetics

## Abstract

**Background/Objectives:** Poly(lactic-co-glycolic acid) (PLGA) microspheres offer sustained drug delivery but often suffer from broad particle size distribution (PSD), leading to inconsistent release profiles. This study investigates wet sieving as a post-processing strategy to precisely control PSD, quantified by the Span value, and evaluates its impact on the performance of triamcinolone acetonide (TA)-loaded PLGA microspheres. **Methods:** Triamcinolone acetonide-loaded PLGA microspheres were prepared via emulsification-solvent evaporation. Wet sieving was employed as a post-processing strategy to obtain distinct particle size fractions and groups with defined polydispersity (Span values). The microspheres were characterized for particle size distribution, drug loading, surface morphology, and in vitro release kinetics. To establish the in vivo relevance of polydispersity control, the pharmacokinetic profiles of different Span groups were first determined using LC-MS/MS following intra-articular injection in rats. Subsequently, their therapeutic efficacy was evaluated in a rat model of knee osteoarthritis, with outcomes assessed by joint swelling measurement and histopathological analysis. **Results:** Microspheres were prepared, fractionated into distinct size groups (0–20, 20–28, 28–40, 40–50, >50 μm) and polydispersity groups (Span = 1.4, 0.8, 0.5). We identified Span as a dominant factor independent of mean particle size. Reducing the Span from 1.4 to 0.5 significantly decreased burst release (24.15% to 14.51%), prolonged mean residence time (MRT 88.52 to 123.53 h), and enhanced anti-inflammatory and cartilage-protective effects in a rat model of knee osteoarthritis. **Conclusions:** This work establishes Span ≤ 0.5 as a critical quality attribute and presents wet sieving as a simple, effective method to ensure batch-to-batch consistency and predictable in vivo performance for PLGA microsphere products.

## 1. Introduction

Microsphere refers to the drug dispersed or dissolved in polymer materials, forming a micron-sized skeletal spherical or globular solid, with a common particle size of 1–250 μm. It has the advantages of controllable drug release, good biocompatibility, and low toxicity and side effects [1]. Poly (lactic-co-glycolic acid) (PLGA) is the most widely studied biodegradable synthetic polymer material in the field of microspheres. In vivo, PLGA is hydrolyzed at the ester bond to lactic acid and glycolic acid, and further metabolized to water and carbon dioxide, which are excreted from the body [2].

It is well-established that the mean particle size of PLGA microspheres is a critical determinant of their drug loading and release kinetics. Pioneering work by Berkland et al. demonstrated that increasing microsphere diameter slows release rates and prolongs release duration [3]. Similarly, Chen et al. reported significant differences in the performance of gefitinib-loaded PLGA microspheres across broad size fractions (e.g., 0–20 vs. 50–100 μm) [4]. Consequently, controlling mean particle size is a central tenet of microsphere formulation. However, in practice, a batch of microspheres is never monodispersed. It possesses a particle size distribution (PSD), typically quantified by the polydispersity index or Span value. While uniform (monodisperse) microspheres are theoretically ideal for predictable release [5,6], the systematic investigation of how PSD independent of mean size affects microsphere properties remains surprisingly limited. This gap is critical because PSD is not merely a statistical descriptor; it directly influences the population heterogeneity of release pathways, potentially leading to unpredictable burst release and variable in vivo performance. Yet, this parameter is often overlooked in quality control.

This study employs triamcinolone acetonide (TA), a potent glucocorticoid, as a model drug for intra-articular therapy of knee osteoarthritis (KOA)-a prevalent and debilitating condition characterized by synovitis and cartilage degradation [7,8,9,10]. While effective, conventional TA suspensions offer only short-term relief [11,12]. Sustained-release PLGA microspheres, exemplified by the FDA-approved product Zilretta^®^ (FX006), present a promising solution by prolonging joint residence [13]. The commercial product is manufactured via spray-drying [14], whereas the simpler emulsion-solvent evaporation method is widely used in research and development. A common challenge with the latter method, however, is the generation of a broad PSD [15], which, as argued above, may undermine release predictability and therapeutic consistency. Therefore, establishing strategies to understand and control PSD is of paramount importance for the development of robust generic or novel PLGA microsphere formulations.

Therefore, this study was designed to systematically deconvolute the effects of PSD on PLGA microsphere performance and to propose a practical control strategy. We hypothesized that the width of the PSD (Span value) is an independent critical quality attribute (CQA) that dictates drug release kinetics and in vivo efficacy, beyond the influence of mean particle size. To test this, we utilized wet sieving as a versatile tool to: (1) isolate microspheres of similar mean size but differing Span values, allowing for the isolation of polydispersity as the sole variable; and (2) obtain distinct size fractions from the same batch to assess the impact of subtle size differences. The effects were rigorously evaluated through comprehensive in vitro characterization, pharmacokinetics, and efficacy studies in a rat model of KOA. Ultimately, this work aims to provide a quantifiable guideline (e.g., a target Span threshold) and a feasible process intervention (wet sieving) to ensure consistent and predictable performance of PLGA microsphere-based drug products.

## 2. Materials and Methods

### 2.1. Materials, Reagents, and Animals

TA (98.30%, Hubei Huizepu Pharmaceutical Technology Co., Ltd., Wuhan, China), DG-75DLGH055 PLGA (acidic end, LA/GA ratio 75/25, IV = 0.45 dL/g, Mw = 54,000 g/mol, Jinan Daigang Bioengineering Co., Ltd., Jinan, China), 1788 Polyvinyl Alcohol (PVA, alcoholysis degree 87–89%, Shanghai Aladdin Biochemical Technology Co., Ltd., Shanghai, China), TA (Reference Standard, 99.64%, Sichuan Puxiao Reference Material Technology Co., Ltd., Chengdu, China), Betamethasone (BMT, Reference Standard, 99.60%, Hefei Xiyan Biotechnology Co., Ltd., Hefei, China).

This experiment utilized male SD rats (Anhui Medical University Experimental Animal Center), weighing 180–200 g, with No. permit SYXK (Wan) 2022-004. All procedures strictly complied with Anhui Medical University Animal Research Ethics Committee requirements (ethical approval number: No. 20242524, approval Date: 21 February 2025). All experiments were carried out on animals in accordance with the Guide for the Care and Use of Laboratory Animals (8th edition, National Academies Press, Washington, DC, USA), and the ARRIVE (Animal Research: Reporting of In Vivo Experiments) guidelines.

### 2.2. Preparation and Screening of Microspheres

Take PLGA (1500 mg) and TA (600 mg) and dissolve them in 10mL solvent (85% dichloromethane (DCM) and 15% *N,N*-dimethylformamide (DMF)) as the organic phase. The organic phase was added dropwise into the PVA solution (50 mL, 5 mg/mL, aqueous phase), with simultaneous emulsification performed for 3 min at 21,200 rpm using a high-speed shear mixer (IKA T10, Germany, Aika Company, Karlsruhe, Germany) at 21,200 rpm for 3 min [16,17]. The resulting emulsion was transferred to 1000 mL of aqueous phase and stirred magnetically at 300 rpm for 4 h [14,18,19]. The solvent was evaporated, and the microspheres were cured. The harvested microspheres were washed three times with copious amounts of ultrapure water (≥50 mL per wash) to remove residual PVA and organic solvents (DCM and DMF). An appropriate amount of lyophilized microspheres was weighed and uniformly dispersed in a PVA solution (5 mg/mL), then sieved through metal meshes with apertures of 50, 40, 28, and 20 μm to obtain microspheres in the size fractions of >50, 40–50, 28–40, 20–28, and 0–20 μm. In addition, the unscreened microspheres were passed through 50 μm and 20 μm sieves to obtain 20–50 μm microspheres. The average particle sizes of the microspheres in the unscreened group, 20–50 μm group and 28–40 μm group were similar, whereas their dispersion properties were different.

### 2.3. Characterization of Microspheres

#### 2.3.1. Particle Size and Distribution

The particle size and polydispersity of the microspheres were determined by laser diffraction using a Mastersizer 3000 particle size analyzer (Malvern Instruments Ltd., Malvern, UK) equipped with a Hydro EV wet dispersion unit. Sample preparation: A small amount of microspheres was gently dispersed in 1 mL of an aqueous solution containing 0.1% (*w*/*v*) Tween-20 to prevent aggregation. The dispersion was then added to the measurement cell containing circulating distilled water until an appropriate obscuration (8–12%) was achieved [20]. Measurement parameters: The refractive index of PLGA is 1.59, while that of the dispersant (water) is 1.33. The absorption index was set to 0.01. Each measurement consisted of three replicates, with at least 10,000 scans per replicate. Data analysis: The particle size distribution was calculated using the instrument’s software (Mastersizer 3000) based on the Mie scattering theory. The following parameters were recorded and are reported in Supplementary Materials Table S1:

*D* [3,4]: The volume-weighted mean diameter (De Brouckere mean). Span value: The width of the distribution, calculated as:

$$Span=\frac{Dv(90)-Dv(10)}{Dv(50)}$$

*Dv(10), Dv(50), Dv(90)*: The particle diameters at which 10%, 50%, and 90% of the volume distribution lie below, respectively. A lower Span value indicates a narrower, more monodisperse distribution [21,22]. Data are presented as the mean ± standard deviation (SD) of three independent measurements (*n* = 3).

#### 2.3.2. Drug Loading

Chromatographic conditions: High Performance Liquid Chromatography system (HPLC-20AD, Shimadzu Corporation, Kyoto, Japan), detector as ultraviolet detector, detection wavelength at 254 nm, chromatographic column as Hypersil ODS 2 column (4.6 × 250 mm, 5 μm), mobile phase as water/acetonitrile (68:32), flow rate at 1.5 mL/min, column temperature at 30 °C, injection volume of 20 µL [23]. Performing a linear regression analysis on the peak area *A* and concentration *C*, we obtain the regression equation: *A* = 24146*C*-332.42 (*R*^2^ = 0.9999), which shows a good linear relationship within the concentration range of 0.5~25 μg·mL^−1^. Accurately weigh 10.0 mg of TA microspheres and place them in a 10 mL volumetric flask. Acetonitrile was added to dissolve the microspheres via ultrasonic treatment, followed by dilution to the marked volume with the mobile phase. The resulting solution was filtered through a 0.22 μm organic filter membrane and finally injected into the instrument for detection.

$$DL\%=\frac{m1}{m2}\times 100\%$$

where *DL%* is the drug loading of microspheres, *m1* is the weight of drug in microspheres (mg), and *m2* is the weight of microspheres (mg).

#### 2.3.3. Microsphere External Morphology

Microspheres were attached to the sample stage coated with conductive adhesive, sputtered with gold, and then observed using a Gemini SEM 300 scanning electron microscope (SEM, Carl Zeiss, Oberkochen, Germany) to obtain sample images.

### 2.4. In Vitro Release Study

#### 2.4.1. In Vitro Release Determination

Add 4.0 mg of TA microspheres to a 25 mL PBS (pH 7.2, containing 0.3% SDS) solution, place in a 35.0 ± 0.5 °C air bath shaker, and shake at a rate of 75 ± 3 rpm [24]. At the predetermined time, the mixture was taken out and centrifuged at 4000 rpm for 10 min. 0.5 mL of the supernatant was removed, an equal volume of PBS was added to the remaining mixture and mixed well, and then the in vitro release was continued. The removed supernatant was mixed with 0.5 mL of mobile phase to prepare the sample solution for the determination of TA content.

Determine the TA content and calculate the cumulative release rate according to the following formula [25]:

$$Q=\frac{{[C}_{n}\times V+V_{0}\times (C_{1}+C_{2}+C_{3}+\cdots +C_{\left(n-1\right)})]}{F\times W\times 1000}\times 100\%$$

where *Q* is the cumulative release degree of microspheres outside the body, *C* is the concentration of drug in the release medium (μg/mL), *V* is the volume of release medium (mL), *V_0_* is the sampling volume (mL), *W* is the weight of microspheres (mg), and *F* is the drug loading of microspheres (%).

The phenomenon where a microsphere formulation rapidly releases a large amount of drug within a day is called ‘burst release’, and the degree of burst release is usually assessed by the cumulative release percentage of the drug over the first 24 h [26]. According to the 2025 edition of the Chinese Pharmacopoeia, the drug content released by the drug-loaded microspheres within the first 0.5 h should be less than 40% [27].

#### 2.4.2. Research on the Release Mechanism

The in vitro drug release data (cumulative release percentage-time curve) were fitted using OriginPro 2024 software and analyzed with Zero-order, First-order, Higuchi, and Ritger-Peppas models. The equations are shown in Table 1 [28].

In addition, this experiment uses the similarity factor method (*f_2_*) to evaluate the similarity of in vitro release curves between different groups of microspheres. The calculation formula is as follows [24]:

$$f_{2}\;=\;50\;\times \;\log{[1\;+\;(\frac{1}{n})\sum_{t=1}^{n}{\left(R_{t}-T_{t}\right)}^{2}]}^{-0.5}\;\times \;100$$

where *Rt* and *Tt* represent the cumulative release percentages at time point t for the control group and the comparison group. *n* is the number of test points. An *f_2_* value between 50 and 100 indicates that the two release curves are similar, whereas a value between 0 and 50 indicates a statistically significant difference in their dissolution profiles [29].

### 2.5. Animal Research

#### 2.5.1. Construction of Knee Arthritis Model

**Figure 1:**
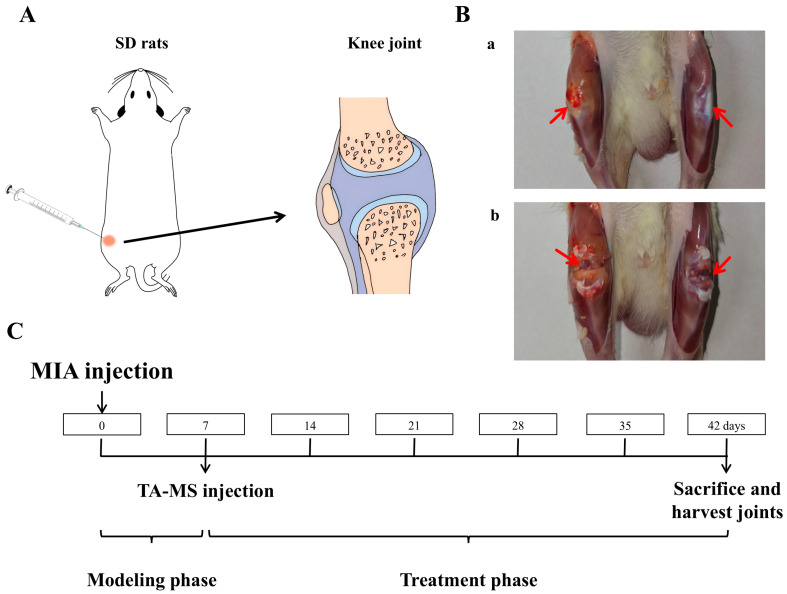
TA microspheres for the study of knee arthritis in rats: (**A**) is a schematic diagram of the injection into the rat knee joint, (**B**) is an effect diagram of the modeling of knee arthritis (a: Knee joint after skin incision, b: Joint cavity after incision, comparison between the right knee with arthritis and the healthy left knee), and (**C**) is the timeline of animal research.

Anesthetize the rat with ether, shave off the hair at the knee joint area, fix it in a supine position, and use an insulin syringe to inject 50 μL of the sodium iodoacetate solution (MIA, in a NaCl solution, 60 mg/mL) into the right knee joint cavity (Figure 1A) [30,31]. Seven days later, it was found that the diameter of the right knee joint had significantly increased. After killing the rats, cut open the skin at the knee joint as shown in Figure 1B(a), and compared with the non-modeled side, it can be seen that the joint on the modeled side is significantly swollen and yellow, after cutting open both joint cavities Figure 1B(b), compared with the non-modeled side, the cartilage is noticeably yellow and relatively rough, indicating a successful modeling of the KOA model.

#### 2.5.2. Grouping and Dosing Regimen

Prepare an isotonic sterile aqueous solution containing sodium chloride (NaCl, 0.9% *w*/*w*), carboxymethyl cellulose sodium (CMC, 0.5% *w*/*w*), and polysorbate−80 (0.1% *w*/*w*) as the microsphere solvent [24]. Different polydispersity microspheres (unsieved, 20–50 μm and 28–40 μm) were weighed separately, added to the solvent, and vortexed to evenly disperse them, serving as the dosage formulation. All batches of microspheres used in animal experiments were strictly screened to ensure that their chemical composition (PLGA type, drug content) and average particle size were essentially consistent, with only significant differences in polydispersity coefficient. This experimental design can exclude the interference from mean particle size and other physicochemical factors, thereby clearly demonstrating the independent effects of polydispersity on in vivo pharmacodynamics and pharmacokinetics.

After adaptation, rats were randomly divided into groups: (1) Normal group, (2) Model group, (3) Unsieved group, (4) 20–50 µm group, and (5) 28–40 µm group. In addition to the normal group, all groups were injected with 50 µL of sodium iodoacetate solution (60 mg/mL) into the joint cavity of the right knee. The normal group was injected with an equal amount of saline. After 7 days of normal feeding, the normal group and model group were injected with 100 μL of solvent into the right joint cavity, while the microsphere administration groups were injected with 100 µL of microsphere suspension, respectively. Based on the body surface area (BSA) conversion principle between humans and rats and the clinical dose of 40 mg for commercially available formulations, assuming an adult body weight of 70 kg, the dosing amount in rats is approximately 6.3 times that of adults, resulting in an equivalent rat dosage of 3.6 mg/kg. After the experiment, rats were euthanized, and their knee joints were dissected, decalcified, and sectioned. Then, H&E, S&F, and immunohistochemical staining were performed (the section staining experiment was entrusted to Anhui Ketu Biotechnology Co., Ltd., Hefei, China, for completion). The timeline is shown in Figure 1C.

### 2.6. Pharmacokinetic Research

#### 2.6.1. Determination of TA Concentration in the Blood

Chromatographic conditions [32,33]: The chromatographic column is Zorbax Eclipse XDB C18 (3.0 × 50 mm, 1.8 μm), mobile phase: A phase is a 2 mM ammonium acetate solution (adjusted to pH 3.2 with formic acid), B phase is acetonitrile, gradient elution, from 0–1.7 min, 45%B, from 1.71–2.0 min, 90%B, from 2.01–3.5 min, 90%B, from 3.51–3.6 min, 45%B, from 3.61–4.5 min, 45%B, flow rate is 0.4 mL/min, column temperature is 40 °C, sample room temperature is 4 °C, injection volume is 5 µL.

Mass spectrometry conditions: Ion source: Electrospray ionization source, positive ion mode detection, Ion spray voltage: 3500 V, Ion source temperature: 350 °C, Vaporization temperature: 300 °C, Sheath gas flow rate: 35 Arb, Auxiliary gas flow rate: 10 Arb. The scan mode is Multiple Reaction Monitoring (MRM), the collision energies for TA and BMT are 14 V and 7 V, respectively.

Perform linear regression with TA concentration X as the horizontal axis and the peak area ratio of TA to BMT Y as the vertical axis, with the standard curve equation: Y = 0.0056*X + 0.0028 (r^2^ = 0.9936). The linear relationship between TA and concentration is good within the range of 0.5~200 ng·mL^−1^.

#### 2.6.2. Collection and Processing of Plasma Samples

After administration, blood samples of 0.3 mL were collected from the rat retrobulbar venous plexus at 0.5, 6, 12, 24, 72, 120, 168, 336, 504, 672, and 840 h into anticoagulant centrifuge tubes, centrifuged at 3000 rpm for 15 min to separate plasma. Plasma samples were processed by the protein precipitation method [32], and their TA content was determined using BMT as the internal standard. Simply, take 100 µL of plasma sample and add BMT acetonitrile solution (400 µL, 250 ng/mL), vortex mix for 1.5 min, then centrifuge at 4 °C, 12,000 rpm for 10 min. Finally, transfer the supernatant, filter it through a 0.22 μm organic filter membrane, and inject it for analysis.

#### 2.6.3. Pharmacokinetic Parameter Calculation and Analysis

Pharmacokinetic data were processed with DAS 2.0 Pharmacokinetic Software, and relevant pharmacokinetic parameters were derived via the statistical moments method based on a non-compartmental model—including maximum plasma concentration (Cmax), time to peak (Tmax), area under the plasma concentration-time curve (AUC), and mean residence time (MRT).

### 2.7. Pharmacodynamic Research

#### 2.7.1. Measurement of Knee Joint Diameter

With the knee bent, measure the distance from the medial to the lateral skin along the direction of the collateral ligament using a vernier caliper [31]. The measurement time is as shown in Figure 1C, where the model side and healthy knee diameter difference values are measured and calculated every 7 days.

#### 2.7.2. Assessment of Synovial Morphology

Rat knee joint sections were stained with H&E and observed and scored using a slide scanner (Pannoramic SCAN, Budapest, Hungary, 3DHISTECH). Krenn scoring criteria [34]: Chronic synovitis was graded and semi-quantitatively assessed based on three characteristics: thickening of the lining cell layer, density of synovial stromal cells, and leukocyte infiltration.

#### 2.7.3. Assessment of Cartilage Tissue Morphology

Slice S&F staining and scoring by slide scanner. OARSI scoring criteria [35]: For the sections stained with Safranin O and Fast Green of each group of rats, the observation and scoring area should include the following four parts of the joint surface (articular cartilage): medial femoral condyle, lateral femoral condyle, medial tibial plateau, and lateral tibial plateau. The scores for each part are summed up to obtain the final score.

#### 2.7.4. Assessment of Inflammatory Factors

Sections were processed for immunohistochemistry (IHC) to detect TNF-α, IL-1β, and IL-6. ImageJ 1.54k was used for grayscale analysis to measure average optical density and for semi-quantitative comparisons between groups.

### 2.8. Statistical Processing

All experiments were repeated at least three times (n ≥ 3). The experimental data are presented as mean ± standard deviation (Mean ± SD) and analyzed using GraphPad Prism 10.1.2. Comparisons between groups were conducted using one-way ANOVA, with statistically significant differences indicated by *p* values < 0.05.

## 3. Results

### 3.1. Microspheres of Different Particle Sizes

#### 3.1.1. Effect of Particle Size Fraction on Drug Loading

**Figure 2:**
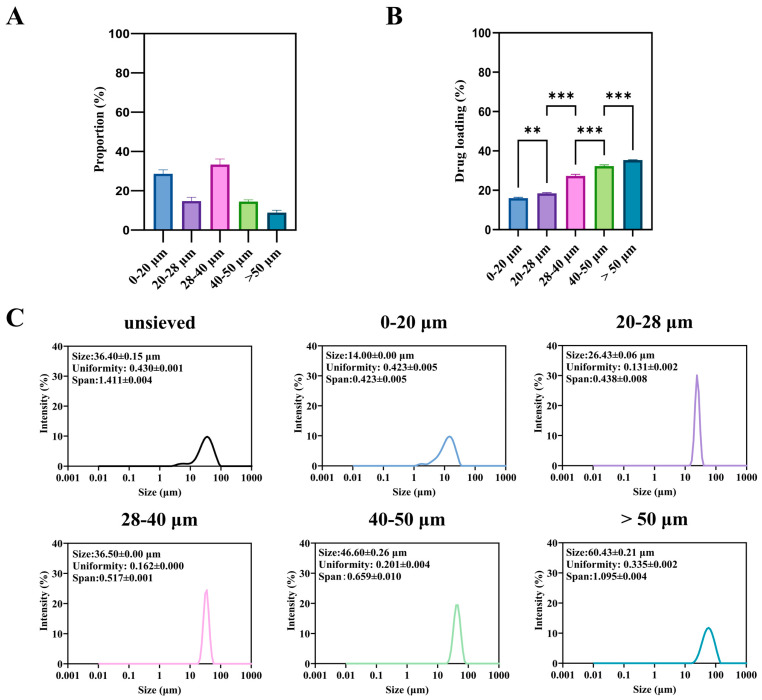
Microspheres of different sizes: (**A**) is the proportion of microspheres of different sizes in the unsieved group, (**B**) is the drug loading and encapsulation rate, and (**C**) is the particle size and distribution. (**: *p* < 0.01, ***: *p* < 0.001).

The unsieved TA-PLGA microspheres exhibited a broad particle size distribution spanning from approximately 2 to 90 μm (Figure 2A). Wet sieving successfully fractionated this bulk material into five distinct subpopulations: 0–20, 20–28, 28–40, 40–50, and >50 μm, whose individual size distributions are shown in Figure 2C.

A significant and monotonic relationship was observed between particle size fraction and drug loading (DL) (Figure 2B). The DL increased progressively from the smallest (0–20 μm) to the largest (>50 μm) fraction. For instance, the DL of the >50 μm fraction was approximately 2.2-fold higher than that of the 0–20 μm fraction (*p* < 0.001). This trend is consistent with previous reports on other drug-PLGA systems [4]. It can be attributed to two primary factors during the emulsion-solvent evaporation process: (1) shorter diffusion paths for both the drug (TA) and the water-miscible solvent (DMF) in smaller droplets, leading to more rapid drug loss to the aqueous phase prior to polymer solidification; and (2) the larger surface area-to-volume ratio of smaller particles, which amplifies this loss.

From a product quality perspective, this inherent variability presents a major challenge. A batch containing a high proportion of small microspheres will not only have a lower overall DL but, as shown in the following section, will also be predisposed to rapid, uncontrollable drug release (burst effect). Therefore, controlling and minimizing the fraction of sub-20 μm particles is not merely a statistical concern but a critical aspect of ensuring consistent drug content and predictable release kinetics. The wet sieving process itself resulted in a distribution of mass yields across the different size fractions (Figure 2A), which must be considered for any potential industrial application.

#### 3.1.2. Morphological Characteristics

**Figure 3:**
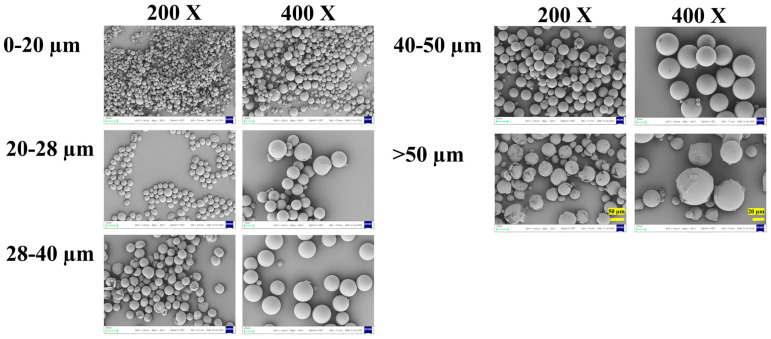
The surface SEM of microspheres with different diameters. 200× SEM images: 50 μm scale bar (left panel, >50 μm group); 400× SEM images: 20 μm scale bar (right panel, >50 μm group).

Scanning electron microscopy (SEM) revealed distinct morphological features among the different size fractions (Figure 3). All microspheres were generally spherical with relatively smooth surfaces, and no major pores or defects were observed at the initial state.

Notably, microspheres in the smaller size fractions (0–20 μm and 20–28 μm) exhibited more perfect sphericity and uniform shape. In contrast, microspheres in the larger fractions (40–50 μm and >50 μm) showed a higher incidence of surface irregularities, partial fragmentation, and occasional agglomeration. This can likely be attributed to the greater susceptibility of larger emulsion droplets to shear-induced deformation and coalescence during the high-speed emulsification and solvent evaporation stages.

These morphological observations have direct implications for process and product quality. The superior sphericity of smaller particles may contribute to better injectability and suspension stability. Conversely, the irregular shape and fragility of some larger particles could pose challenges for reproducible manufacturing and handling, potentially leading to batch-to-batch variability. Furthermore, the physical integrity of the microsphere matrix, as inferred from surface smoothness, is a prerequisite for achieving the designed release profile, which will be explored in the next section.

#### 3.1.3. In Vitro Release Kinetics and Mechanisms

##### Release Profiles and Burst Release

**Figure 4:**
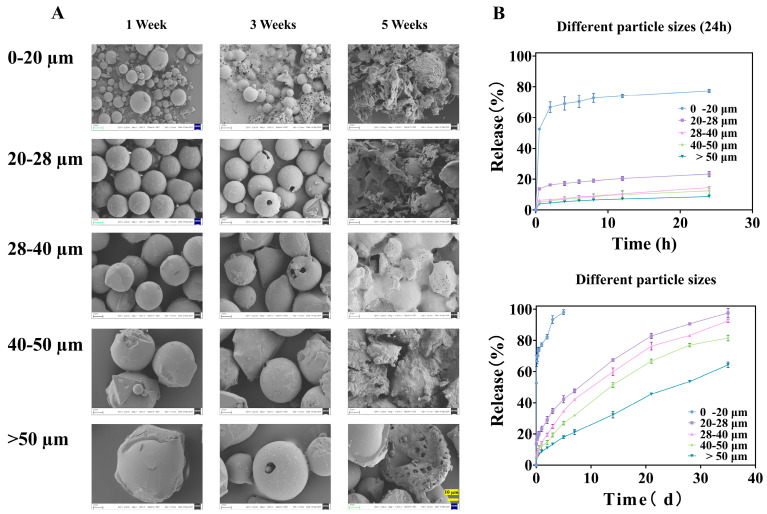
Microspheres of different particle sizes: (**A**) is the SEM image released at different times, all magnified 1000× (The ruler is uniformly set to 10 μm), and (**B**) is the in vitro release curve.

The in vitro release profiles of TA from microspheres of different size fractions exhibited pronounced and systematic differences (Figure 4B). A strong inverse correlation was observed between particle size and release rate. The smallest fraction (0–20 μm) displayed the most rapid release, reaching 52.54 ± 0.31% within the first 0.5 h and nearly complete release (>98%) within 5 days. This pattern is characteristic of a substantial burst release, which far exceeds the 40% threshold suggested for sustained-release formulations in some compendia [36].

The similarity factor (between 0–20 and 20–28 μm groups is 18.69, between 20–28 and 28–40 μm groups is 49.39, between 28–40 and 40–50 μm groups is 49.35, and between 40–50 and >50 μm groups is 32.54) analysis confirmed the statistical dissimilarity between the release profiles of adjacent size fractions (all f2 values < 50), underscoring that even a 10 μm difference in the mean size cut-off can lead to a clinically and pharmaceutically relevant change in release behavior.

##### Release Mechanism Elucidated by Model Fitting

To elucidate the underlying release mechanisms, the data were fitted to various kinetic models (Table 2). The release from the 0–20 μm and 20–28 μm fractions was best described by the Ritger–Peppas model, with release exponents (n) of 0.10 and 0.38, respectively (both < 0.45). This indicates a Fickian diffusion-controlled mechanism, in which drug release is primarily governed by concentration-gradient-driven diffusion through the polymer matrix or pores. This is consistent with the hypothesis that smaller particles have shorter diffusion pathways and a higher surface-area-to-volume ratio, favoring rapid drug egress.

For the larger fractions (28–40, 40–50, and >50 μm), the n values increased to 0.49, 0.51, and 0.58, respectively, falling within the range 0.45 < n < 0.89. This signifies a shift toward anomalous transport or a coupled mechanism involving both diffusion and polymer matrix erosion. The longer diffusion paths in larger particles allow time for the PLGA backbone to hydrolyze, thereby influencing the release kinetics.

##### Morphological Evolution During Release

SEM images captured at different time points during the release study provided visual corroboration of these mechanisms (Figure 4A). After one week, all microspheres maintained their spherical shape but developed surface roughness, indicating initial surface erosion/drug depletion. By week three, significant degradation was evident only in the 0–20 μm fraction, which began to agglomerate and lose structural integrity. The larger fractions showed only minor pitting or cracking. After five weeks, the 0–20 μm microspheres had completely disintegrated into fragments, while the larger particles, though heavily eroded, largely retained their particulate structure. This direct visualization confirms that smaller microspheres degrade much more rapidly, which, together with diffusion, accelerates drug release.

##### Implications for Formulation Design and Quality Control

The collective findings from Section 3.1.1, Section 3.1.2 and Section 3.1.3 paint a coherent picture: Smaller TA-PLGA microspheres are associated with lower drug loading, faster degradation, and a dominant diffusion-driven burst release. From a product development standpoint, this is critical. The presence of even a minor subpopulation of small microspheres (e.g., the 0–20 μm fraction visible in the unsieved batch, Figure 2A) can disproportionately dominate the early release phase, leading to unpredictable pharmacokinetics and potential safety concerns (e.g., transient systemic exposure).

Therefore, rigorous control over the particle size distribution, specifically the minimization of the fines (sub-20 μm) fraction, is not optional but essential for developing a reliable sustained-release microsphere product. This provides a strong rationale for employing sieving or other classification techniques as part of a Quality by Design (QbD) strategy to ensure a consistent and desirable release profile.

### 3.2. Microspheres with Different Polydispersity

#### 3.2.1. In Vitro Microsphere Characteristics

**Figure 5:**
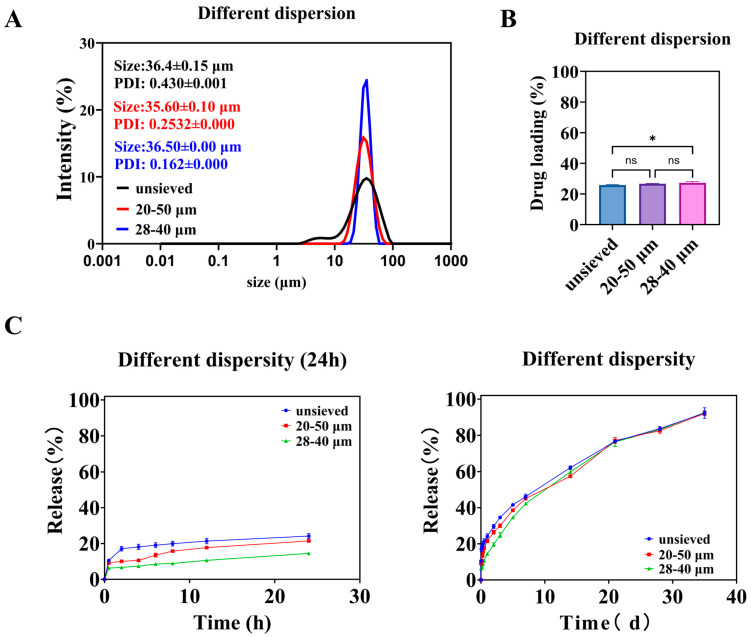
Different polydispersity microspheres: (**A**) is the particle size and distribution of the microspheres, (**B**) is the drug loading of the microspheres, and (**C**) is the in vitro release curve of the microspheres. (*: *p* < 0.05, ns: *p* > 0.05).

As the screening range narrows, the particle size distribution of microspheres becomes more concentrated. As shown in Figure 5A, microspheres with an average diameter of about 36 μm and Span values of 1.4, 0.8, and 0.5 can be obtained through screening. The drug loading among the groups of microspheres was similar (Figure 5B), except for the unsieved group, which had significantly different drug loading compared to the 28–40 μm group of microspheres. As described in Section 3.1.1, microspheres with small particle sizes have a high proportion but low drug loading, which has a minor impact. Microspheres with large particle sizes have a high drug loading but a low proportion, which also has a minor impact. At the same time, the most significant proportion of unsieved microspheres is the 28–40 μm part. After screening, microspheres with different dispersities have the same core parts. Therefore, when the dispersion of microspheres with a value of 1.4 is reduced to 0.8, it is essentially the same, whereas when it is reduced to 0.5, there is a significant difference of drugloading.

#### 3.2.2. In Vitro Release

As shown in Figure 5C, the release of microspheres over a day decreases with decreasing polydispersity. During the early stages of release, the release from the unsieved group was consistently higher than that from the 20–50 μm group, and the release from the 20–50 μm group was consistently higher than that from the 28–40 μm group. Combining the in vitro release curves of microspheres with different particle sizes (Figure 2), it can be seen that small-sized microspheres are the main cause of early release. The 28–40 μm microsphere group has the fewest small-sized microspheres and the slowest release rate. The similarity comparison of the in vitro release curves for three groups of microspheres showed that the similarity factor between the unsieved group and the 20–50 μm group was 72.57, between the unsieved group and the 28–40 μm group was 58.70, and between the 20–50 μm group and the 28–40 μm group was 65.30. The similarity factors among them exceeded 50, indicating a certain similarity in their dissolution profiles.

The fitting results for the in vitro cumulative release data are shown in Table 2. In the Ritger–Peppas exponents, the *n* values for the unsieved and 20–50 μm groups were 0.35 and 0.41, respectively, both below 0.45, indicating that the release process is primarily controlled by diffusion. The n value for the 28–40 μm group was 0.49, falling between 0.45 and 0.89, suggesting that the release process is governed by both diffusion and matrix erosion. The difference in n values likely arises from the broader particle size distribution in the unsieved 20–50 μm group, which contains a higher proportion of smaller microspheres that exhibit faster, diffusion-dominated release.

#### 3.2.3. The Independent Role of Polydispersity: Isolating the Effect of Span Value

##### Design Rationale and Microsphere Characteristics

To isolate the specific effect of particle size distribution (polydispersity) from that of mean particle size, we employed a two-step wet sieving strategy on the same bulk preparation. This yielded three groups with similar volume-weighted mean diameters (D [3,4]) around 36 μm but distinctly different polydispersity indices: “Unsieved” (Span ≈ 1.4), “20–50 μm” (Span ≈ 0.8), and “28–40 μm” (Span ≈ 0.5) (Figure 5A, see exact values in Supplementary Table S1). This design ensures that any observed differences in performance can be attributed primarily to the width of the distribution (Span) rather than to a shift in the central tendency.

The drug loading (DL) across these three groups was statistically similar (Figure 5B), confirming the success of the experimental design in controlling the mean size and composition. The slightly lower DL in the unsieved group is likely attributable to its contained proportion of very small particles (0–20 μm), which, as established in Section 3.1.1, have inherently lower DL.

##### Span Value Dictates Burst Release and Release Profile

The in vitro release profiles revealed a striking dependence on Span value (Figure 5C). The extent of burst release (24 h cumulative release) decreased monotonically as Span decreased: it was highest for the unsieved group, intermediate for the 20–50 μm group, and lowest for the 28–40 μm group. This trend directly implicates the presence of fine particles as the primary driver of early burst release, as narrowing the distribution (reducing Span) effectively removes these tails from the population.

The similarity factor (f2) analysis indicated that the profiles of the three groups were similar (f2 > 50), but with a clear trend. The greatest similarity was between the two sieved groups (20–50 μm vs. 28–40 μm, f2 = 65.30), while the unsieved group differed more from the narrowest distribution group (f2 = 58.70). This suggests that improving uniformity (reducing Span) modifies the release curve in a predictable and continuous manner.

##### Mechanism: How Span Value Modulates Release Kinetics

Fitting results of in vitro drug release are shown in Table 2. The Ritger–Peppas exponents for unsieved and 20–50 μm microspheres were 0.35 and 0.41, respectively, suggesting the drug release was mainly dominated by diffusion. In contrast, the 28–40 μm group (Span = 0.5) had an n value of 0.49, suggesting a transition toward a coupled diffusion-erosion mechanism.

This mechanistic shift can be explained by the increased homogeneity of the microsphere population. A narrower distribution means a more uniform diffusion path length and more synchronized polymer degradation across all particles. As the proportion of “outlier” fast-releasing small particles diminishes, the collective release behavior becomes less dominated by simple diffusion and more reflective of the intrinsic erosion-controlled release of the predominant, similarly sized particles.

##### The Span Threshold Concept and Implications for Quality Control

The data point to a critical threshold effect. While reducing Span from 1.4 to 0.8 yielded measurable improvements, the most significant qualitative and quantitative changes in release kinetics (reduced burst, mechanistic shift) occurred when Span reached approximately 0.5. This value represents a point where the particle population becomes sufficiently uniform to fundamentally alter its collective release behavior.

Therefore, for TA-PLGA microspheres of this composition and size range, controlling Span to ≤0.5 emerges as a tangible and impactful quality target. Achieving this target, for instance, via the demonstrated wet sieving process, would directly address one of the most common challenges in PLGA microsphere development: excessive and variable burst release. This establishes Span not just as a descriptive parameter, but as a true Critical Quality Attribute (CQA) that can be actively controlled to ensure a desired product performance.

### 3.3. Pharmacokinetic Study: Translating Polydispersity Control into Predictable Systemic Exposure

#### 3.3.1. Plasma Concentration–Time Profiles: From Burst to Sustenance

**Figure 6:**
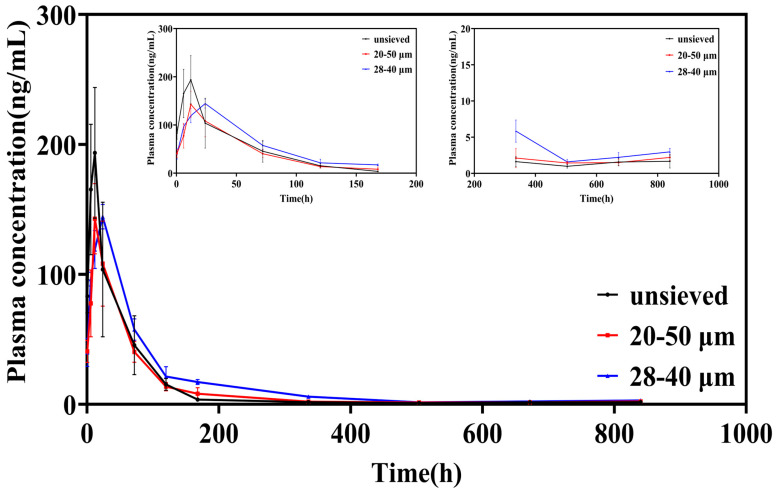
Blood concentration-time curve of TA in SD rats after intra-articular injection of TA microspheres (n = 6, Mean ± SD).

The plasma concentration-time profiles following intra-articular injection starkly reflected the in vitro release characteristics (Figure 6). Microspheres from the unsieved group (Span ≈ 1.4) produced a sharp, high peak (Cmax = 216.97 μg/L) within a short time (Tmax = 10 h), followed by a rapid decline. This pattern is the in vivo hallmark of significant burst release, leading to a transient, high systemic exposure.

In contrast, both sieved groups exhibited attenuated and delayed peaks. The Cmax was significantly lower (149.87 and 144.32 μg/L for the 20–50 μm and 28–40 μm groups, respectively), and the Tmax was prolonged to 14 h and 22 h, respectively. Notably, after the initial phase, the plasma concentrations for the sieved groups, particularly the 28–40 μm group (Span = 0.5), were sustained at higher levels than the unsieved group from approximately 120 h onwards. This indicates a more consistent and prolonged drug supply from the more uniform microsphere populations.

#### 3.3.2. Key Pharmacokinetic Parameters: Quantifying the Benefits of Narrow Distribution

Analysis of the non-compartmental pharmacokinetic parameters provided quantitative evidence of superior sustained-release performance with reduced polydispersity (Table 3).

Peak Exposure (C_max_): The ~33% reduction in Cmax for the 28–40 μm group compared to the unsieved group is clinically significant, as it directly translates to a lower risk of systemic corticosteroid-related side effects.

Mean Residence Time (MRT): The MRT increased from 88.52 h (unsieved) to 123.53 h (28–40 μm), representing a ~40% extension. This parameter confirms that the drug remains in the body (and by extension, is likely released locally at the joint) for a substantially longer duration, favoring sustained therapeutic action.

Area Under the Curve (AUC): The AUC values in the 28–40 μm group exhibited an increasing trend. Although these values were not always statistically significantly different from those in the 20–50 μm group, this trend indicated that the microspheres in this group had a more uniform particle size, thus resulting in more complete drug release. A uniform particle size may enhance the bioavailability of the drug at the injection site.

#### 3.3.3. In Vitro–In Vivo Correlation (IVIVC) and Mechanistic Coherence

The pharmacokinetic findings are in direct mechanistic coherence with the in vitro data. The high initial Cmax and short Tmax for the unsieved group correlate with its high in vitro burst release. Conversely, the lower Cmax, longer Tmax, and extended MRT for the low-Span groups align with their reduced burst and more sustained in vitro release profiles.

This qualitative level A IVIVC strengthens the premise that controlling the in vitro release through polydispersity adjustment reliably predicts and controls the in vivo pharmacokinetic behavior. The shift from a high, sharp peak to a lower, broader profile upon reducing Span from 1.4 to 0.5 is a direct pharmacokinetic manifestation of the suppressed burst release and more synchronized drug release achieved by a narrow particle size distribution.

#### 3.3.4. Implications for Product Development: Safety, Efficacy, and Consistency

The observed pharmacokinetic improvements have direct implications for the therapeutic profile of a microsphere product. A lower and delayed peak (C_max_, T_max_) enhances the safety margin by minimizing peak-related systemic adverse events. A prolonged MRT supports sustained local efficacy by maintaining drug presence.

Therefore, the pharmacokinetic data provide a powerful rationale for stringent control over microsphere polydispersity. Achieving a low Span value (e.g., ≤0.5) is not merely a cosmetic specification; it is a formulation strategy to engineer a more desirable pharmacokinetic profile-one that balances sustained efficacy with reduced systemic exposure. This solidifies the status of Span as a Critical Quality Attribute (CQA) with direct clinical relevance, as its control directly dictates key pharmacokinetic outcomes that impact both safety and effectiveness.

### 3.4. Therapeutic Efficacy of Microspheres with Different Polydispersity in a Rat KOA Mode

#### 3.4.1. Polydispersity-Dependent Amelioration of Joint Swelling and Inflammation

**Figure 7:**
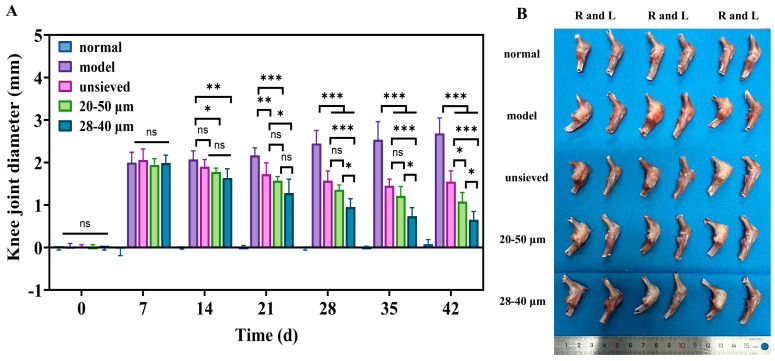
(**A**) The difference in joint diameter between the right and left knee (n = 6), (**B**) The morphology of the isolated rat knee after treatment for 5 weeks, when sacrificed (n = 3). *: *p* < 0.05, **: *p* < 0.01, ***: *p* < 0.001, ns: *p* > 0.05.

**Figure 8:**
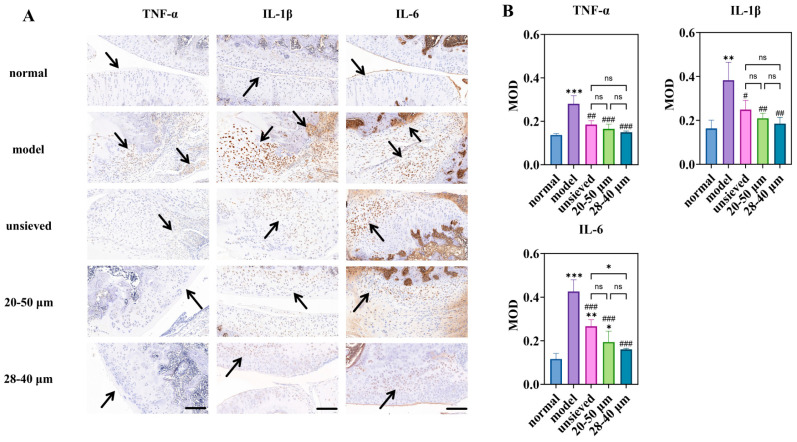
Immunohistochemical study of microspheres with different polydispersity: (**A**) is the immunohistochemical staining for inflammatory factors (magnification 40×, scale bar 200 μm), (**B**) is a semi-quantitative analysis of optical density values (MOD) for IHC sections (n = 3). * Indicates the comparison between each group and the normal group, *: *p* < 0.05, **: *p* < 0.01, ***: *p* < 0.001, # Indicates the comparison between each group and the model group, #: *p* < 0.05, ##: *p* < 0.01, ###: *p* < 0.001. (ns: *p* > 0.05, Arrows point to the prominent positive staining in the sections).

The therapeutic impact of TA microspheres on KOA symptoms was profoundly influenced by their polydispersity. Joint swelling, a primary clinical indicator of inflammation, showed a clear negative correlation with Span value (Figure 7A). While all TA-treated groups showed a significant reduction in swelling compared to the model group, the 28–40 μm group (Span ≈ 0.5) exhibited the fastest and most pronounced resolution. This visual and metric improvement was mechanistically explained at the molecular level by immunohistochemical analysis of key inflammatory cytokines (TNF-α, IL-1β, IL-6) (Figure 8).

Notably, the expression of IL-6, a pivotal driver of synovitis and cartilage catabolism in OA, displayed a strict polydispersity-dependent inhibition (Figure 8B). The IL-6 levels in the unsieved group were comparable to those in the 20–50 μm group, while they were significantly higher than those in the 28–40 μm microsphere group. This result is critical: it demonstrates that achieving a sufficiently narrow particle distribution (Span ≤ 0.5) enables a more complete and sustained local anti-inflammatory effect, effectively “normalizing” the pathological joint microenvironment. The superior control over IL-6 by the low-Span microspheres provides a direct cellular and molecular rationale for their observed superior efficacy in alleviating joint swelling.

#### 3.4.2. Cartilage and Synovium Protection Correlates with Distribution Uniformity

**Figure 9:**
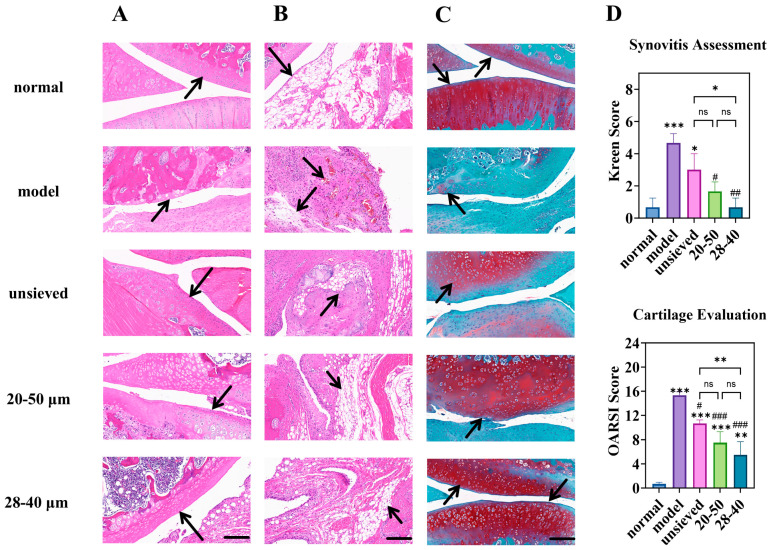
Knee joint section: H&E staining of (**A**) (cartilage) and (**B**) (synovium), (**C**) is the Toluidine Blue O staining (magnification 40×, scale bar is 200 µm), (**D**) is the semi-quantitative analysis of H&E and Toluidine Blue O staining (n = 3). * Indicates the comparison between each group and the normal group, *: *p* < 0.05, **: *p* < 0.01, ***: *p* < 0.001, # Indicates the comparison between each group and the model group, #: *p* < 0.05, ##: *p* < 0.01, ###: *p* < 0.001. (ns: *p* > 0.05, Arrows point to the prominent features of the synovium and cartilage in the sections).

Histopathological evaluation of joint tissues provided definitive evidence of disease modification. In the model group, severe cartilage erosion, loss of proteoglycan (indicated by faint Safranin O staining), and marked synovial hyperplasia were evident (Figure 9A–C). All TA microsphere treatments mitigated these pathologies, but the degree of protection was inversely proportional to the polydispersity.

The microspheres with the narrowest distribution (28–40 μm, Span = 0.5) afforded the most robust cartilage preservation, evidenced by deeper Safranin O staining, more intact surface structure, and significantly lower OARSI scores (Figure 9C,D). Similarly, synovitis scores (Krenn score) decreased progressively from the unsieved to the 28–40 μm group (Figure 9D). This coherent trend across multiple independent histological scoring systems confirms that microsphere population uniformity translates directly into more homogeneous and effective drug exposure at the tissue level. Pharmacodynamic results demonstrated that regulating Span enables modulation of the microsphere release rate and optimization of the drug release timeline, thereby achieving a more effective treatment for chronic and progressive osteoarthritis.

#### 3.4.3. Integrated Efficacy Analysis: Linking Physical Attribute to Biological Outcome

Collectively, the pharmacodynamic data present a compelling and consistent narrative: superior control over microsphere polydispersity yields superior therapeutic outcomes in a complex disease model. The gradient of efficacy—from unsieved (Span ≈ 1.4) to 20–50 μm (Span ≈ 0.8) to 28–40 μm (Span ≈ 0.5)—mirrors the gradient of improved in vitro release and pharmacokinetic profiles established in preceding sections.

This establishes a direct cause-and-effect relationship between the physical quality attribute (Span), the resulting drug exposure profile (prolonged MRT, lower C~max~), and the ultimate biological endpoints (reduced swelling, lower cytokine levels, tissue preservation). The fact that a simple, measurable physical parameter can predict and dictate such a multifaceted in vivo response powerfully validates its status as a Critical Quality Attribute (CQA) with direct clinical relevance. These efficacy findings seamlessly integrate with and substantiate the pharmacokinetic conclusions, setting the stage for a holistic discussion on the implications for product design.

## 4. Study Limitations and Future Perspectives

It is important to note some limitations of the current study. First, the residual levels of the co-solvent DMF in the final microsphere product were not quantified. While DMF is highly water-miscible and extensive washing was employed, its complete removal cannot be guaranteed without analytical verification. Future studies should include residual solvent analysis to ensure compliance with safety guidelines (e.g., ICH Q3C). Second, the direct measurement of TA concentration within the joint synovial fluid was not performed, which would provide the most direct evidence of localized sustained release.

## 5. Conclusions

This study systematically decouples the effects of particle size and particle size distribution on the performance of triamcinolone acetonide (TA)-loaded PLGA microspheres, moving beyond the conventional focus on mean diameter. By employing wet sieving as an effective tool, we have demonstrated that particle size distribution, quantified by the Span value, is an independent and dominant Critical Quality Attribute (CQA) that governs drug loading, release kinetics, pharmacokinetics, and ultimately, therapeutic efficacy in a model of knee osteoarthritis.

Our key findings establish a clear hierarchy of control: First, the fraction of fine particles (e.g., <20 μm) must be minimized, as they contribute disproportionately to low drug loading and high burst release. Second, and more importantly, for microspheres of similar mean size, reducing the polydispersity (Span value) directly translates into superior product performance. We identified a target Span threshold of approximately 0.5, below which burst release is significantly suppressed, mean residence time is extended, and anti-inflammatory/cartilage-protective effects are maximized. The wet sieving process presented herein serves as a practical and proven strategy to achieve this narrow distribution, offering a straightforward path to improve batch-to-batch consistency.

The implications of this work are twofold. For formulation scientists, it provides a quantitative framework for Quality by Design (QbD): Span value should be monitored and controlled as a key parameter during development and production. For regulatory and quality control, it highlights the necessity of including polydispersity, not just average size, in the specifications of microsphere-based drug products to ensure predictable clinical performance.

While this study provides a robust in vitro-in vivo correlation, we acknowledge that further investigation into intra-articular drug concentrations and the long-term fate of the polymer carrier would provide a more complete picture. Nevertheless, the compelling link established between a simple physical attribute (Span), controllable release profiles, and enhanced in vivo outcomes offers a powerful blueprint for the development of next-generation, reliable sustained-release microsphere therapies.

In addition to the significant impact of particle size and its distribution on the in vitro and in vivo properties of triamcinolone acetonide PLGA microspheres, the formulation recipe and preparation process, as fundamental factors regulating the core quality attributes of the microspheres, also play a crucial role. The ratio of lactic acid to glycolic acid in PLGA within the prescription composition, along with its molecular weight, directly determines the degradation rate and sustained-release cycle of the microspheres [37,38]. Relevant studies have confirmed that these formulation parameters affect the release kinetics and biological safety of the formulation by controlling the microsphere encapsulation rate, morphological structure, and drug dispersion [38,39].

## Figures and Tables

**Table 1:** In vitro drug release fitting equations.

Kinetic Model	Equation
*Zero-order*	*Q = k_1_t*
*First order*	*ln(100 − Q) = ln100 − _2_t*
*Higuchi*	*Q = k_3_t_1/2_*
*Ritger–Peppas*	*Q_t_/Q_∞_ = k_4_t_n_*

*Q* represents the cumulative release percentage at time t, *k* is the model rate constant, and *R^2^* is the correlation coefficient. The diffusion exponent *n* in the *Ritger–Peppas* model is used to determine the release mechanism: *A* value of 0.45 < *n* < 0.89 indicates *non-Fick* diffusion (i.e., the combined action of diffusion and erosion), *n* > 0.89 approaches zero-order release, and *n* < 0.45 indicates *Fickian* diffusion.

**Table 2:** Analysis of the kinetic fitting results of the external release of TA microspheroids.

Group	Model	Zero-Order	First-Order	Higuchi	Ritger–Peppas
0–20 µm	R^2^	0.7839	0.3237	0.9029	0.9449
20–28 µm		0.9417	0.8417	0.9960	0.9790
28–40 µm		0.9338	0.9738	0.9959	0.9957
40–50 µm		0.9302	0.9802	0.9888	0.9889
>50 µm		0.9738	0.9737	0.9844	0.9910
unsieved		0.9288	0.8325	0.9967	0.9854
20–50 µm		0.9311	0.9098	0.9968	0.9927

**Table 3:** Pharmacokinetic Characterization of TA Microspheres in SD Rats (n = 6, Mean ± SD).

Parameter	Unit	Unsieved	20–50 μm	28–40 μm
T_max_	h	10.00 ± 3.1	14.00 ± 4.9	22.00 ± 4.9 ***^#^
C_max_	ug/L	(2.17 ± 0.25)*10^2^	(1.50 ± 0.24)*10^2^ ***	(1.44 ± 0.090)*10^2^ ***
AUMC_(0−t)_		(8.81 ± 2.3)*10^5^	(10.26 ± 2.1)*10^5^	(16.75 ± 1.7)*10^5^ ***^###^
MRT_(0−t)_	h	(0.89 ± 0.15)*10^2^	(1.07 ± 0.13)*10^2^	(123.53 ± 9.0)***
AUC_(0−t)_	ug/L*h	(10.20 ± 3.1)*10^3^	(9.60 ± 1.9)*10^3^	(13.55 ± 0.76)*10^3^ *^#^
AUC_(0−∞)_	ug/L*h	(11.14 ± 3.1)*10^3^	(10.96 ± 1.9)*10^3^	(14.23 ± 1.0)*10^3^ *^#^

* *p* < 0.05 vs. Unsieved, *** *p* < 0.001 vs. Unsieved, # *p* < 0.05 vs. 20–50 μm, ### *p* < 0.001 vs. 20–50 μm.

## Data Availability

The data presented in this study are available on request from the corresponding author due to the raw/processed data required to reproduce these findings cannot be shared at this time as the data also forms part of an ongoing study.

## Supplementary Materials

The following supporting information can be downloaded at: https://www.mdpi.com/article/10.3390/pharmaceutics18020180/s1. Figure S1: XRD spectra of unsorted microspheres and raw materials; Table S1: Particle size distribution of TA microspheres; Table S2: The effect of different mixed solvents on TA microspheres; Table S3: The effect of different PVA concentrations and varying oil-to-water volume ratios on TA microspheres.

## Abbreviations

The following abbreviations are used in this manuscript:

PLGA	Poly(lactic-co-glycolic acid)
PSD	Particle size distribution
TA	Triamcinolone acetonide
CQA	Critical quality attribute
BMT	Betamethasone
DCM	Dichloromethane
DMF	N,N-Dimethylformamide
PVA	Polyvinyl Alcohol
DL	Drug loading

## References

[1] Rahimi M., Charmi G., Matyjaszewski K., Banquy X., Pietrasik J. (2021). Recent developments in natural and synthetic polymeric drug delivery systems used for the treatment of osteoarthritis. Acta Biomater..

[2] Bee S.-L., Hamid Z.A.A., Mariatti M., Yahaya B.H., Lim K., Bee S.-T., Sin L.T. (2018). Approaches to Improve Therapeutic Efficacy of Biodegradable PLA/PLGA Microspheres: A Review. Polym. Rev..

[3] Berkland C., King M., Cox A., Kim K., Pack D.W. (2002). Precise control of PLG microsphere size provides enhanced control of drug release rate. J. Control. Release.

[4] Chen W., Palazzo A., Hennink W.E., Kok R.J. (2017). Effect of Particle Size on Drug Loading and Release Kinetics of Gefitinib-Loaded PLGA Microspheres. Mol. Pharm..

[5] Vlachopoulos A., Karlioti G., Balla E., Daniilidis V., Kalamas T., Stefanidou M., Bikiaris N.D., Christodoulou E., Koumentakou I., Karavas E. (2022). Poly(Lactic Acid)-Based Microparticles for Drug Delivery Applications: An Overview of Recent Advances. Pharmaceutics.

[6] Tran V.-T., Benoît J.-P., Venier-Julienne M.-C. (2011). Why and how to prepare biodegradable, monodispersed, polymeric microparticles in the field of pharmacy?. Int. J. Pharm..

[7] Katz J.N., Arant K.R., Loeser R.F. (2021). Diagnosis and treatment of hip and knee osteoarthritis: A review. JAMA.

[8] Gelber A.C. (2024). Knee osteoarthritis. Ann. Intern. Med..

[9] Giorgino R., Albano D., Fusco S., Peretti G.M., Mangiavini L., Messina C. (2023). Knee osteoarthritis: Epidemiology, pathogenesis, and mesenchymal stem cells: What else is new? An update. Int. J. Mol. Sci..

[10] Richard M.J., Driban J.B., McAlindon T.E. (2023). Pharmaceutical treatment of osteoarthritis. Osteoarthr. Cartil..

[11] Stout A., Friedly J., Standaert C.J. (2019). Systemic absorption and side effects of locally injected glucocorticoi ds. PM&R.

[12] McAlindon T.E., LaValley M.P., Harvey W.F., Price L.L., Driban J.B., Zhang M., Ward R.J. (2017). Effect of intra-articular triamcinolone vs saline on knee cartilage volume and pain in patients with knee osteoarthritis. JAMA.

[13] Kraus V.B., Conaghan P.G., Aazami H.A., Mehra P., Kivitz A.J., Lufkin J., Hauben J., Johnson J.R., Bodick N. (2018). Synovial and systemic pharmacokinetics (PK) of triamcinolone acetonide (TA) following intra-articular (IA) injection of an extended-release microsphere-based formulation (FX006) or standard crystalline suspension in patients with knee osteoarthritis (OA). Osteoarthr. Cartil..

[14] Bodick N., Blanks R.C., Kumar A., Clayman M.D., Moran M. Corticosteroids for the Treatment of Joint Pain. TW-201219039-A, 2017. https://pubchem.ncbi.nlm.nih.gov/patent/TW-201219039-A.

[15] Dang Y., Guan J. (2020). Nanoparticle-based drug delivery systems for cancer therapy. Smart Mater. Med..

[16] Park H. (2024). Exploring the Effects of Process Parameters during W/O/W Emulsion Preparation and Supercritical Fluid Extraction on the Protein Encapsulation and Release Properties of PLGA Microspheres. Pharmaceutics.

[17] Molavi F., Barzegar-Jalali M., Hamishehkar H. (2022). Changing the daily injection of glatiramer acetate to a monthly long acting product through designing polyester-based polymeric microspheres. Bioimpacts.

[18] Doty A.C., Zhang Y., Weinstein D.G., Wang Y., Choi S., Qu W., Mittal S., Schwendeman S.P. (2017). Mechanistic analysis of triamcinolone acetonide release from PLGA microspheres as a function of varying in vitro release conditions. Eur. J. Pharm. Biopharm..

[19] Doty A.C., Hirota K., Olsen K.F., Sakamoto N., Ackermann R., Feng M.R., Wang Y., Choi S., Qu W., Schwendeman A. (2016). Validation of a cage implant system for assessing in vivo performance of long-acting release microspheres. Biomaterials.

[20] Li K. (2020). Preparation, Evaluation and Pharmacodynamics of Dopamine Receptor Agonist Extended-Release Microspheres. Ph.D. Thesis.

[21] D’Souza S., Faraj J.A., Dorati R., DeLuca P.P. (2015). Enhanced degradation of lactide-co-glycolide polymer with basic nucleophilic drugs. Adv. Pharm..

[22] Burin G.R.M., Santos T.C.d., Battisti M.A., Campos A.M.d., Ferreira S.R.S., Carciofi B.A.M. (2022). Transport properties of hydrophilic compounds in PLGA microspheres. Res. Soc. Dev..

[23] Tipnis N.P., Shen J., Jackson D., Leblanc D., Burgess D.J. (2020). Flow-through cell-based in vitro release method for triamcinolone acetonide poly (lactic-co-glycolic) acid microspheres. Int. J. Pharm..

[24] Flexion Therapeutics, Inc Drug Approval Package: ZILRETTA (Triamcinolone Acetonide) [Package Insert]. U.S. Food and Drug Administration. https://www.accessdata.fda.gov/drugsatfda_docs/nda/2017/208845Orig1s000TOC.cfm.

[25] Wang L., Duan T., Gao Y., Liu H., Sun R., Wu T., Tang J. (2025). Development and in vitro-in vivo evaluation of a novel sustained-release tablet based on constant-release surface. J. Drug Delivery Sci. Technol..

[26] Schutzman R., Shi N.-Q., Olsen K.F., Ackermann R., Tang J., Liu Y.-Y., Hong J.K.Y., Wang Y., Qin B., Schwendeman A. (2023). Mechanistic evaluation of the initial burst release of leuprolide from spray-dried PLGA microspheres. J. Control. Release.

[27] Kolasinski S.L., Neogi T., Hochberg M.C., Oatis C., Guyatt G., Block J., Callahan L., Copenhaver C., Dodge C., Felson D. (2020). 2019 American college of rheumatology/arthritis foundation guideline for the management of osteoarthritis of the hand, hip, and knee. Arthritis Care Res..

[28] Lee H.G., Park Y.S., Jeong J.H., Kwon Y.B., Shin D.H., Kim J.Y., Rhee Y.S., Park E.S., Kim D.W., Park C.W. (2019). Physicochemical properties and drug-release mechanisms of dual-release bilayer tablet containing mirabegron and fesoterodine fumarate. Drug Des. Devel. Ther..

[29] Xie F., Ji S., Cheng Z. (2015). In vitro dissolution similarity factor (f2) and in vivo bioequivalence criteria, how and when do they match? Using a BCS class II drug as a simulation example. Eur. J. Pharm. Sci..

[30] Chun J.M., Lee A.Y., Nam J.Y., Lim K.S., Choe M.S., Lee M.Y., Kim C., Kim J.-S. (2021). Effects of Dipsacus asperoides Extract on Monosodium Iodoacetate-Induced Osteoarthritis in Rats Based on Gene Expression Profiling. Front. Pharmacol..

[31] Kwon M., Nam D., Kim J. (2023). Pathological Characteristics of Monosodium Iodoacetate-Induced Osteoarthritis in Rats. Tissue Eng. Regen. Med..

[32] Ho M.J., Jeong H.T., Im S.H., Kim H.T., Lee J.E., Park J.S., Cho H.R., Kim D.Y., Choi Y.W., Lee J.W. (2019). Design and In Vivo Pharmacokinetic Evaluation of Triamcinolone Acetonide Microcrystals-Loaded PLGA Microsphere for Increased Drug Retention in Knees after Intra-Articular Injection. Pharmaceutics.

[33] César I.C., Byrro R.M., de Santana e Silva Cardoso F.F., Mundim I.M., de Souza Teixeira L., de Sousa W.C., Gomes S.A., Bellorio K.B., Brêtas J.M., Pianetti G.A. (2011). Determination of triamcinolone in human plasma by a sensitive HPLC-ESI-MS/MS method: Application for a pharmacokinetic study using nasal spray formulation. J. Mass Spectrom..

[34] Krenn V., Morawietz L., Burmester G.R., Kinne R.W., Mueller-Ladner U., Muller B., Haupl T. (2006). Synovitis score: Discrimination between chronic low-grade and high-gra de synovitis. Histopathology.

[35] Pritzker K.P.H., Gay S., Jimenez S.A., Ostergaard K., Pelletier J.-P., Revell P.A., Salter D., van den Berg W.B. (2006). Osteoarthritis cartilage histopathology: Grading and staging. Osteoarthr. Cartil..

[36] Chinese Pharmacopoeia Commission (2025). Chinese Pharmacopoeia (Part II).

[37] Kohno M., Andhariya J.V., Wan B., Bao Q., Rothstein S., Hezel M., Wang Y., Burgess D.J. (2020). The effect of PLGA molecular weight differences on risperidone release from microspheres. Int. J. Pharm..

[38] Wan B., Bao Q., Burgess D. (2023). Long-acting PLGA microspheres: Advances in excipient and product analysis toward improved product understanding. Adv. Drug Deliv. Rev..

[39] Hua Y., Su Y., Zhang H., Liu N., Wang Z., Gao X., Gao J., Zheng A. (2021). Poly(lactic-co-glycolic acid) microsphere production based on quality by design: A review. Drug Deliv..

