# Supplementary material for: Span Value as a Critical Quality Attribute for PLGA Microspheres: Controlling Burst Release and Enhancing Therapeutic Efficacy via Wet Sieving
Source: Pharmaceutics. 2026 Jan 30;18(2):180. doi: 10.3390/pharmaceutics18020180 (PMC12944294; doi:10.3390/pharmaceutics18020180)
Supplement: Supplementary file 1 [file pharmaceutics-18-00180-s001.zip › pharmaceutics-4097358-supplementary.pdf]

Supplementary Figure S1

XRD spectra of unsorted microspheres and raw materials.

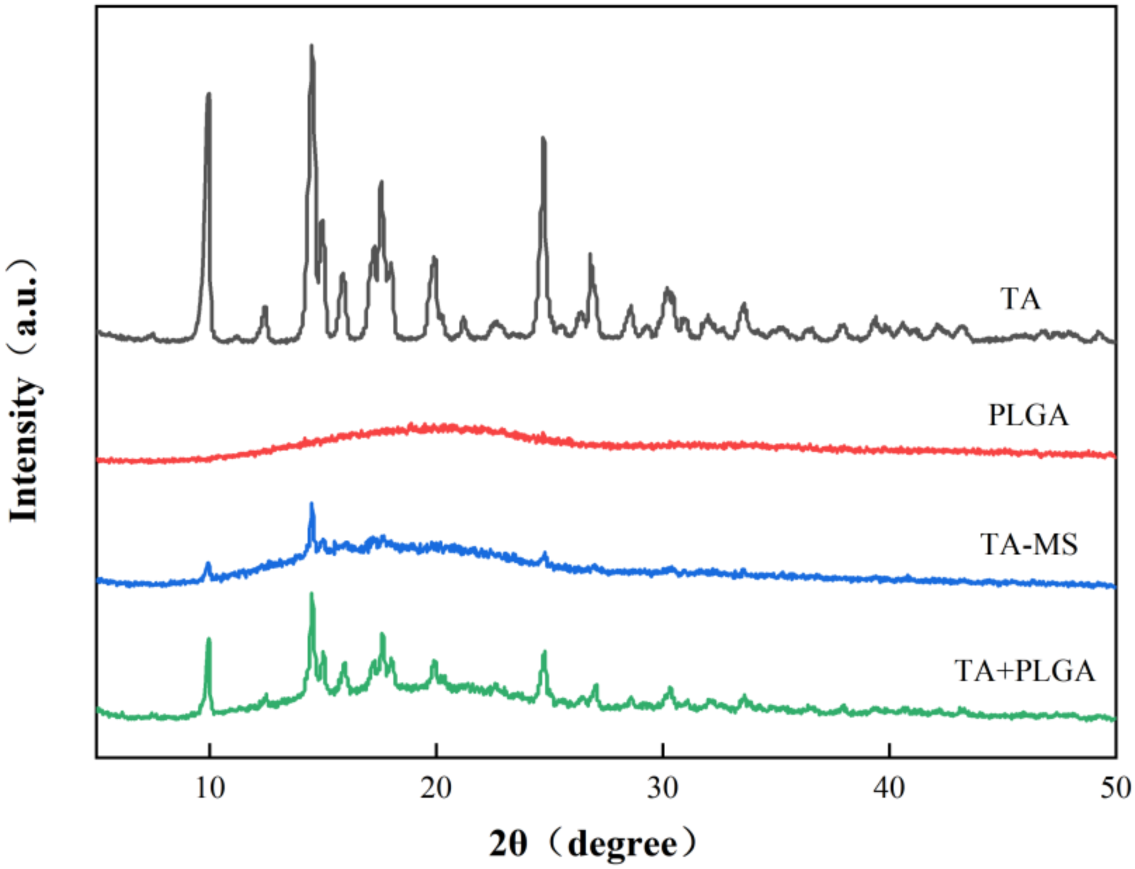

Instrument name: X-ray diffractometer (DX-2700BH, Liaoning Dandong Haoyuan Instrument Co., Ltd.).

Methods: The presence of TA in the samples was determined by observing the changes in peak shape and intensity using X-ray diffraction (XRD) patterns. A suitable amount of TA, PLGA, TA+PLGA, and TA-MS was uniformly pressed onto a sample holder for XRD analysis. The scanning parameters were as follows: step size 0.02, range 5-50, tube current 30 mA, tube voltage 40 kV, scanning rate 5/min, and X-ray source: Cu target.

As shown in the figure, pure TA and TA+PLGA (a physical mixture of TA and PLGA) frequently exhibit crystalline diffraction peaks including 9.9, 14.5, 15,15.9, 17.6, 19.9, and 24.7 within the test range, indicating the crystalline state of TA. In contrast, PLGA does not display clear and intense diffraction peaks, and TA-MS almost never shows drug-related diffraction peaks (combined with SEM images, the occasional appearance of diffraction peaks may be related to minor microsphere fragmentation). This not only confirms the amorphous state of PLGA but also demonstrates that TA is effectively loaded onto the PLGA matrix and exists in an amorphous or molecular state.

Supplementary Table S1

Particle size distribution of TA microspheres. Data are expressed as the means ± SD (n = 3).

Size Group (μm)	d(4,3) (μm)	d10 (μm)	d50 (μm)	d90 (μm)	Span	Uniformity
0-20	14.00 ± 0.00	5.29 ± 0.09	13.30 ± 0.00	23.70 ± 0.10	1.387± 0.016	0.423 ± 0.005
20-28	26.43 ± 0.06	21.10 ± 0.10	26.10 ± 0.00	32.57 ± 0.12	0.438 ± 0.008	0.131 ± 0.002
28-40	36.50 ± 0.00	27.70 ± 0.00	35.90 ± 0.00	46.23 ± 0.06	0.517 ± 0.001	0.162 ± 0.000
40-50	46.60 ± 0.26	32.67 ± 0.12	45.40 ± 0.10	62.57 ± 0.55	0.659± 0.010	0.201 ± 0.004
50-	64.47 ± 0.23	34.87 ± 0.15	60.43 ± 0.21	101.00 ± 0.00	1.095 ± 0.004	0.335 ± 0.002
Unsieved	36.43 ± 0.15	14.13 ± 0.06	34.20 ± 0.10	62.37 ± 0.31	1.411 ± 0.004	0.430 ± 0.001
20-50	35.60 ± 0.10	22.73 ± 0.06	34.00 ± 0.10	50.63 ± 0.15	0.821 ± 0.001	0.252 ± 0.000

The span value was calculated with the following formula: Span = (d90 − d10)/d50, where d90 is the particle diameter at 90% cumulative size, d10 is the particle diameter at 10% cumulative size and d50 is the particle diameter at 50% cumulative size. The d(4,3) is a commonly used average particle size parameter in laser particle size analysis. Its full name is Volume-weighted mean diameter; it is also known as De Brouckere mean diameter or Volume-fourth-moment mean diameter. In the reports or software of microgranularity analyzers (such as laser particle size analyzers), “Uniformity” is a parameter used to describe the breadth of the particle size distribution, indicating whether the particle sizes in the sample are concentrated or consistent.

Based on the preliminary research on TA-MS formulations by the research team, we conducted comparative screening of microsphere formulation conditions. The experimental results are presented in Table 2-3.

For the same batch of microspheres used in the study, the particle size of the microspheres produced using the scaling-up process underwent slight changes, but these changes were within an acceptable range.

Supplementary Table S2

The effect of different mixed solvents on TA microspheres (n=3, Mean±SD)

Solvents	DL%	EE%	IBR%	D(4,3) µm	Span
DCM/DMF(100/0)	23.88±1.01	83.57±3.54	16.41±0.62	81.23±2.40	3.88±0.12
DCM/DMF(85/15)	25.82±0.78	90.37±2.37	25.36±1.77	37.90±0.10	1.34±0.06
DCM/DMF(70/30)	25.30±1.28	88.56±4.49	23.21±0.83	58.40±2.84	1.89±0.07
DCM/DMSO(85/15)	25.17±1.00	88.10±3.49	28.49±1.11	50.40±1.91	1.50±0.01
DCM/AC(85/15)	18.93±0.59	66.27±2.07	19.67±0.71	59.17±2.04	2.41±0.17
DCM/DIO(85/15)	20.46±0.68	71.61±2.39	32.27±1.18	66.37±0.51	3.26±0.02
DCM/EAC(85/15)	21.24±0.34	74.33±1.20	16.66±0.15	73.03±2.01	3.92±0.14

Supplementary Table S3

The effect of different PVA concentrations and varying oil-to-water volume ratios on TA microspheres (n=3, Mean±SD)

		DL%	EE%	IBR%	D(4,3)µm	Span
PVA concentration (mg/mL)	5	25.82±0.78	90.37±2.37	25.36±1.77	37.90±0.10	1.34±0.06
	10	25.48±0.70	89.19±2.45	31.73±0.53	30.67±0.06	1.39±0.02
	20	23.86±0.84	83.51±2.94	40.43±1.63	23.50±2.36	1.53±0.01
V oil/V water	1/5	25.82±0.78	90.37±2.37	25.36±1.77	37.90±0.10	1.34±0.06
	1/10	23.40±0.81	81.91±2.83	28.04±1.19	38.33±0.50	1.55±0.00
	1/20	23.15±0.33	81.05±1.17	20.66±0.65	97.07±1.50	2.40±0.05
